# The effects of surgical preparation techniques and implant macro-geometry on primary stability: An *in vitro* study

**DOI:** 10.4317/medoral.21286

**Published:** 2017-02-04

**Authors:** Giovanni Falisi, Marco Severino, Claudio Rastelli, Sara Bernardi, Silvia Caruso, Massimo Galli, Luca Lamazza, Carlo Di Paolo

**Affiliations:** 1 MD. Department of Life, Health, and Environmental Sciences, School of Dentistry, University of L’Aquila, L’Aquila, Italy; 2 PhD. Department of Life, Health, and Environmental Sciences, School of Dentistry, University of L’Aquila, L’Aquila, Italy; 3 DDS. Department of Life, Health, and Environmental Sciences, School of Dentistry, University of L’Aquila, L’Aquila, Italy; 4 MD Department of Oral and Maxillofacial Sciences, “Sapienza” University of Rome, Italy

## Abstract

**Background:**

The attainment of a good primary stability is a necessary condition to ensure the success of osseointegration in implantology. In type IV cancellous bone, however, it is possible that a reduced primary stability can lead to an increased rate of failure. The aim of this study was therefore to determine, with the help of the resonance frequency (Osstell mentor), which technique of implant site preparation (piezo surgery, conventional, under-preparation, bone compaction, osteodistraction) and macro-geometry is able to improve implant stability in type IV cancellous bone.

**Material and Methods:**

10 pig ribs were prepared with a surgical pre-drilled guide, calibrated for a correct implant positioning. On each rib, 5 implant sites (one for each technique) were prepared. Successively, 50 conical implants (Tekka Global D) were inserted and measured with the resonance frequency to evaluate the primary stability. Data collected were analyzed by analysis of variance (ANOVA) to test whether the Implant Stability Quotient (ISQ) values of the five techniques were significantly different.

**Results:**

The results showed that no significant differences among the ISQ values of the five techniques used were found. Also, no significant differences in the macro-geometry of the two types of compared implants were observed. However, the macro-geometry of Tekka implants, characterized by a double condensing thread, seems to provide greater ISQ values than those of single thread implants when using the same technique.

**Conclusions:**

In light of these preliminary data, it is conceivable that in cases of reduced stability, such as those occurring with a type IV bone, all means ameliorating the primary stability and accelerating the osseointegration can be utilized.

**Key words:**Implant primary stability, resonance frequency analysis, implant site preparation.

## Introduction

Osseointegration is a biological response that leads to a structural direct connection between the vital bone and the surface of an implant under functional load. This process is attributed to a series of sequential activation processes of osteoblasts, with subsequent production and mineralization of the peri-implant osteoid tissue. However, in case of reduced implant stability with micromotions passing the threshold (50-150 nm) or bone necrosis caused by overheating of the cutters, the fibrous encapsulation will overcome the osseointegration (1). Thus, the primary implant stability becomes one of the most important prerequisites to achieve a successful osseointgration and must be obtained already during the surgical phase and maintained through the entire healing period (2).

The primary stability is a mechanical process influenced by several factors related to the implants (design, size, macro and micro surface), the bone substrate (bone quality and quantity) and the operator (surgical technique). Several studies have compared the *in vitro* and *in vivo* improvement of primary stability in conical implants as compared to the cylindrical ones (3,4). The treatment modalities of the implant surface may also ameliorate the primary stability, since an increase in roughness improve the contact between the bone and implant. Moreover, the sandblasted and etched implants promote the osteogenesis by improving osteoblastic activity when compared to implants exclusively machined (5,6).

Regarding bone quantity and quality, there are several studies on the matter because most of the failures in implantology is linked more to the quality than quantity of the bone. Friberg *et al.* already reported a great number of failures in case of resorbed maxillary and soft bone (7). Also Jaffin and Bernam in a retroprospective study had evidenced the failure of implants in patients with poor bone density while other authors documented that the main reason for failure of implants was not linked to the healing process but, on the opposite, to the undeniable influence of reduced bone density with a rate of success ranging from 28 to 65% (8).

In literature some clinical methods for the evaluation of implant stability are described, such as the radiographic method, implant percussion, the perio test evaluation and the contro torque test (9-11). However, these methods lead to obtain results of subjective evaluation or do not allow a linear evaluation of the stability. With the introduction of the resonance frequency, it was possible to switch from a self-interpreting form of evaluation to a real evaluation and linearly correlated with the degree of stability of the implant.

Thus the aim of this study was to evaluate the primary stability of 5 different techniques of implant site preparation using the resonance frequency (Osstell mentor) in a model of type IV animal cancellous bone. Ten pig ribs were prepared with conventional surgical technique (TC); under-preparation technique (TS); technique of bone expanders (TE); bone compactor technique (TO); and technique with the piezo surgery (TP). A total of 50 conical Tekka dental implants (10 implants/technique) were inserted and Implant Stability Quotient (ISQ) was measured with Osstell mentor.

## Material and Methods

In this study, 10 were obtained from the local slaughterhouse. The rib samples measured 7x3x2 cm.

The choice of this type of bone was dictated by its simple availability and by its similarity to the human structure and trabecular composition (12).

Great attention has been given to the selection of the proximal part of the ribs, since the cortical component of this area is structurally reduced. Furthermore, this cortical part was completely eliminated, leaving exposed only the medullar part where the implants would be then located (13).

To obtain a type 4 bone, the sample was further treated with a 20% glacial acetic acid solution at 21°C one hour before starting the experiment. Such procedure is used to increase the decalcification of the trabecular bone (14).

In addition, a Plexiglas reference surgical Jig was prepared to encode the distance between the implants, and 5 reference points were positioned at a distance of 10 mm from each other. This distance has been chosen to abolish possible modifications or interferences produced on the bone during the preparation of the sites.

To decide the sequence of the implant site preparation and increase the statistical rigor, 10 schemes, out of possible 15625 combination, were randomly selected with the help of a computer device.

For this study, 50 conical Tekka dental implants (10 mm high x 3.5 mm diameter), titanium grade 6 TA6V, with truncated cone-shaped screw, progressive double condensing threads and SA2 surface (sandblasted and double acid-attacked) were used. The surface treatment is made by blasting with corundum 250 micron particles followed by a double acid chemical attack. The University of L’Aquila ethical committee approved the publication of this data, reference number 38534.

- Protocol of the surgical techniques:

1) Conventional surgical technique (TC) with dedicated surgical drills, as indicated by the manufacturer with a sequential cutter passage: 1.2 mm pilot drill, 2.2 mm intermediate preparation cutter, 3.2 mm terminal drill, to apply the implants in bones with large cancellous component.

The preparations were performed using a surgical handpiece NSK, with speeds of 800 rpm and torque of 50 newton. The preparations were carried out using a mechanical positioner that would guarantee the same applied pressure and verticality of the drill used.

Thanks to a load cell placed below the implant site area of bone sample, it was possible to maintain the working pressure in a range between 500 and 800 gr. A guide system preformed and calibrated for the different diameters was used to obtain the perpendicular orientation of the cutters with respect to the implant site area of bone sample.

2) Under-preparation technique (TS) with the preparation sequence, pilot drill 1.2 mm, preparation cutter 2.2 mm and terminal cutter 3 mm. The operating parameters described for the previous technique have also been adopted in the under-preparation technique.

For the preparation of the implant site with the conventional and under-preparation techniques, the Branemark protocol with continuous saline irrigation was adopted.

3) Technique of bone expanders (TE): the manufacturer’s instructions were followed, the protocol contemplated to perform the hole until the desired length with an 1.2 mm pilot drill and then the use of bone expanders of various sizes in a sequential manner until reaching the established 3 mm diameter.

4) Bone compactor technique (TO): the Summers protocol was followed, with the preparation sequence: pilot drill and bone compactors n° 1, 2 and 3, to obtain a 3 mm diameter.

5) Technique with the piezo surgery (TP): the preparation of the implant site was made with the sequence of IM1s, IM2, P2-3, IM3 (Mectron Medical Technologies). By using the mechanical positioner and a system of guides preformed, it was possible to perpendicularly orient the inserts and apply controlled pressures. For IM1S the operating pressures were below 150 g, while for IM2, IM3 and P2-3 between 300 and 500 gr. The working movement adopted contemplated the alternation of longitudinal and rotational phases, as well as interruptions as described by before (15).

The piezosurgery was set on the function “SPECIAL” for using IM1S and on the function “IMPLANT” for IM2, P2-3 and IM3. Each insert has been used with continuous irrigation at a speed of 30 ml/min. Also in this technique, the preparations of the implant site have reached a depth of 10mm.

All implants were mechanically inserted with the aid of a handpiece ratio 1/253 at a speed of 50 RPM, 32 N of torque up to the height of preparation of 10mm (16).

In order to be evaluated for the implant stability through the instrument Osstell Mentor (Osstell instrument, Integration Diagnostics AB, Gothenburg, Sweden), a special key, the smart peg 59, with a torque of 10 N has been inserted to each implant. This key has been used for the detection in bidirectional directions (vertical and perpendicular to the rib axis) as specified by the manufacturer (17).

In addition, to evaluate the implant macro-geometry on the primary stability, the ISQ values obtained in a previous work with Maco implants have been compared to those of this experiment (18).

- Statistical analyses

The distribution of the ISQ values was compared to the normal distribution by using three different tests (Kolmogorov-Smirnov, Lilliefors and Shapiro-Wilk). Then, one-way analysis of variance (one-way ANOVA) was used to test the effect of the technique on the *in vitro* implant stability, in other words to explore whether the ISQ values of the five techniques differed in statistically significant manner. Furthermore, the Student’s t-test was used to compare the values of implant stability obtained by the two manufacturers and inserted *in vitro* with the same technique.

## Results

**Figure 1:**
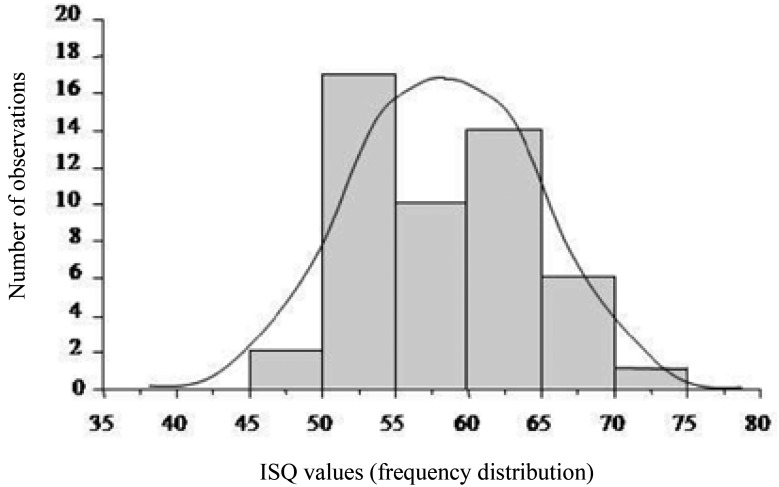
Frequency distribution of ISQ values. ISQ: Implant Stability Quotient.

The distribution of ISQ values was not significantly different from a normal distribution in all three tests, legitimating the use of ANOVA (Fig. 1).

The developed one-way ANOVA model was not statistically significant (f-value: 0.373; *p*-value: 0.826), and no statistically significant difference between the ISQ values corresponding to the various techniques used was found.

**Figure 2:**
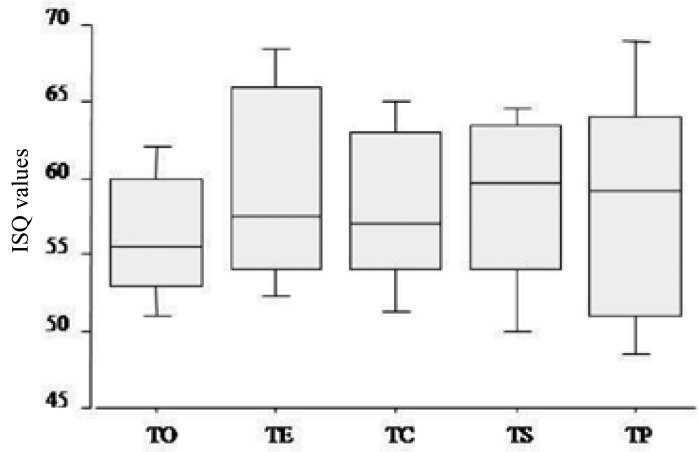
Mean ISQ values of the five techniques. Conventional surgical technique (TC); Under-preparation technique (TS); Technique of bone expanders (TE); Bone compactor technique (TO); Technique with the piezo surgery (TP);; ISQ: Implant Stability Quotient.

As reported in  Table 1, the ISQ values corresponding to the various techniques are very similar, with the possible exception of the TO technique showing a slightly lower stability (Fig. 2).

**Table 1:**
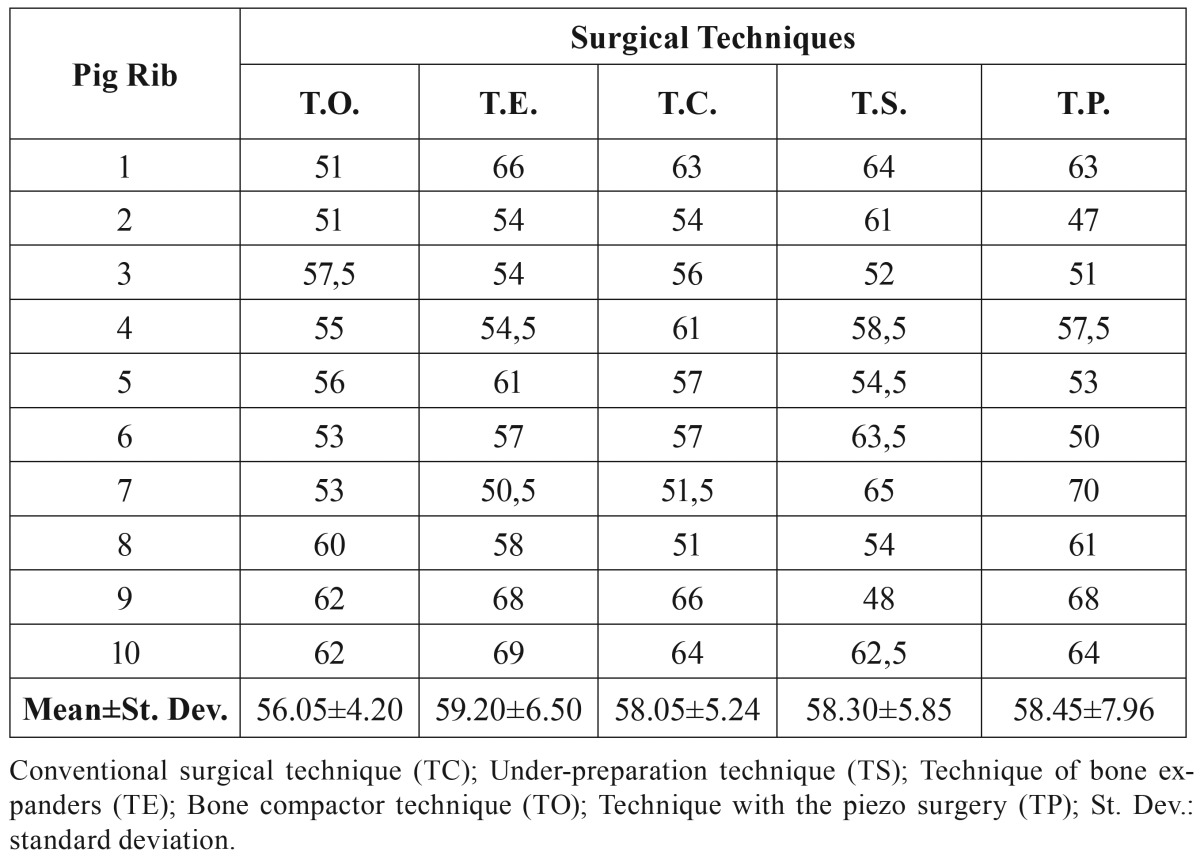
Implant Stability Quotient (ISQ) values of the five techniques.

**Figure 3:**
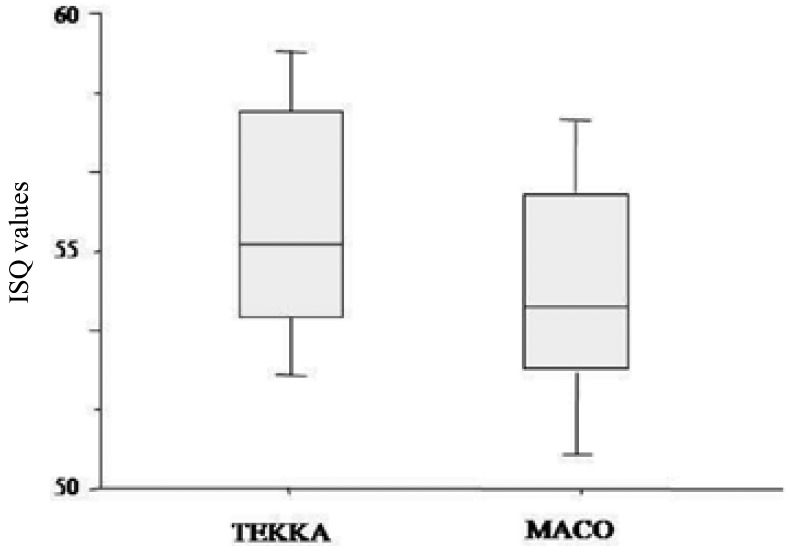
Mean ISQ values of TO for Maco and Tekka implants. Bone compactor technique (TO); ISQ: Implant Stability Quotient.

The t-tests performed thereafter to verify the presence of possible interactive effects between the two explanatory variables showed no statistically significant difference between the Macoand Tekka implant stability (t-value: 0.99; *p*-value: 0.335), regardless of the *in vitro* insertion technique, although the average values in Maco implants are always generally lower (Fig. 3).

## Discussion

Implant stability play a major role in osseointegration. The cortical medullary ratio and thus the bone quality directly affect the primary stability resulting in a direct correlation between cortical thickness and ISQ. Other factors such as the shape of the implant, the surface treatment, as well as the experience of the operator, can directly affect the primary stability (19). In the present study the implant stability of five different techniques of site preparation was evaluated, using implants of predetermined length shape in bone samples of type IV. The results of ISQ measurements performed on 50 implants are comparable, as no statistical significance has emerged among the various techniques performed *in vitro* in bone type IV. Thus, the data obtained with identical insertion torque seem to indicate that the primary stability is not affected by the preparation technique in case of bone deprived of the cortical component and with low mineralization.

The primary stability and the surgical procedure to obtain it have been matter of investigation in numerous studies. Some authors have proposed the technique of dimensional under-preparation of the implant site, in order to improve the bone-implant coupling and thus the implant stability (20,21). Other authors recommend the osseocompaction through osteotomes, to modify the bone density and improve stability (22).

Sethi reported an implant success rate of 97%, with a an adequate implant stability in the superior maxilla using expanders technique. This result is confirmed by Kraft that found a considerable increase in stability and a high insertion torque when compared expanders technique with rotating technique (23).

Other techniques of site preparation, such as piezoelectric technique, were evaluated to determine eventual benefits. The preparation with piezosurgery seems to increase bone density (24) as compared to the conventional technique, although the implant stability is not different from that of the technique with rotating drills (25). However, the piezoelectric technique would favor an improved healing of the implant site in the early stages with a bone remodeling already at 56 days after treatment, as reported previously (26).

Our data show that the 5 techniques look equivalent, despite slight variations in the diameter of the preparation (3 mm for piezosurgery, osteotomes, bone expanders, under-preparation and 3.2 mm for tekka protocol). These results are in line with those reported in literature (25-28). In addition, no statistical differences between Tekka and Maco implants were noted, indicating that the macrogeometry of the manufacturer does not influence implant stability. However, it cannot be excluded that the lack of statistical significance may be due to the relatively small size of the sample analyzed. In particular, it should be noted that Maco implants present a lower average stability than Tekka implants.

With the limitations related to an *in vitro* study, the ISQ values obtained indicate that, regardless of the technique, in bone type IV immediate loading procedures are inadvisable, although a sufficient stability for an osseointegration process is guaranteed (29). As reported by the manufacturer’s instruction for the use of osstell, ISQ values lower than 60 should be considered as low stability, suggesting to monitor over time the successive changes. The lower ISQ values were obtained with osteotomes technique, while the highest values with the technique with bone expanders. This difference could be attributed to the different site of bone compaction, apical and lateral respectively. Probably the greater lateral compaction of the expanders increases the area useful for a suitable contact between bone and implant. In fact, as already described by Martinez, in case of facing a bone with established low density and insufficient stability, it is recommended to use the bicortical anchorage.

In conclusion, it is important to follow simple precautions to improve the implant stability: to use conical implants also active at the neck level, to take advantage of all the anchorage; under prepare the implant site to keep as much as possible the cortical residual; and search for bicorticalism where possible.

In light of the present results, in clinical practice of bone type IV, a technique appears interchangeable with another, since none of them brings advantage to the implant stability. The choice should instead be directed towards the technique that accelerates the processes of healing and osseointegration.

## References

[1] Mellado-Valero A, Ferrer-García JC, Calvo-Catalá J, Labaig-Rueda C (2010). Implant treatment in patients with osteoporosis. Med Oral Patol Oral Cir Bucal.

[2] Mathieu V, Vayron R, Richard G, Lambert G, Naili S, Meningaud JP (2014). Biomechanical determinants of the stability of dental implants: influence of the bone-implant interface properties. J Biomech.

[3] Zonfrillo G, Matteoli S, Ciabattini A, Dolfi M, Lorenzini L, Corvi A (2014). Analysis and comparison of clutch techniques of two dental implants. J Mech Behav Biomed Mater.

[4] Toyoshima T, Tanaka H, Ayukawa Y, Howashi M, Masuzaki T, Kiyosue T (2015). Primary Stability of a Hybrid Implant Compared with Tapered and Cylindrical Implants in an Ex Vivo Model. Clin Implant Dent Relat Res.

[5] Guizzardi S, Galli C, Martini D, Belletti S, Tinti A, Raspanti M (2004). Different titanium surface treatment influences human mandibular osteoblast response. J Periodontol.

[6] Franchi M, Bacchelli B, Giavaresi G, De Pasquale V, Martini D, Fini M (2007). Influence of different implant surfaces on peri-implant osteogenesis: Histomorphometric analysis in sheep. J Periodontol.

[7] Friberg B, Jemt T, Lekholm U (1991). Early failures in 4,641 consecutively placed Brånemark dental implants: a study from stage 1 surgery to the connection of completed prostheses. Int J Oral Maxillofac Implants.

[8] Herrmann I, Lekholm U, Holm S, Kultje C (2005). Evaluation of patient and implant characteristics as potential prognostic factors for oral implant failures. Int J Oral Maxillofac Implants.

[9] Coutant JC, Seguela V, Hauret L, Caix P, Ella B (2014). Assessment of the correlation between implant stability and bone density by computed tomography and resonance frequency analysis in fresh cadavers. Int J Oral Maxillofac Implants.

[10] Jeong MA, Jung MK, Kim SG, Oh JS (2015). Implant Stability Measurements in the Long-Term Follow-up of Dentis Implants: A Retrospective Study With Periotest. Implant Dent.

[11] Gehrke SA, Marin GW (2015). Biomechanical evaluation of dental implants with three different designs: Removal torque and resonance frequency analysis in rabbits. Ann Anat.

[12] Oliscovicz NF, Shimano AC, Marcantonio Junior E, Lepri CP, Dos Reis AC (2013). Analysis of primary stability of dental implants inserted in different substrates using the pullout test and insertion torque. Int J Dent.

[13] Barfeie A, Wilson J, Rees J (2015). Implant surface characteristics and their effect on osseointegration. Br Dent J.

[14] Moon SH, Um HS, Lee JK, Chang BS, Lee MK (2010). The effect of implant shape and bone preparation on primary stability. J Periodontal Implant Sci.

[15] Laurito D, Lamazza L, Garreffa G, De Biase A (2010). An alternative method to record rising temperatures during dental implant site preparation: a preliminary study using bovine bone. Ann Ist Super Sanita.

[16] Valente ML, Lepri CP, dos-Reis AC (2014). In vitro microstructural analysis of dental implants subjected to insertion torque and pullout test. Braz Dent J.

[17] Choi HH, Chung CH, Kim SG, Son MK (2014). Reliability of 2 implant stability measuring methods in assessment of various periimplant bone loss: an in vitro study with the Periotest and Osstell Mentor. Implant Dent.

[18] Rastelli C, Falisi G, Gatto R, Galli M, Saccone E, Severino M (2014). Implant stability in different techniques of surgical sites preparation: an in vitro study. Oral Implantol (Rome).

[19] Rozé J, Babu S, Saffarzadeh A, Gayet-Delacroix M, Hoornaert A, Layrolle P (2009). Correlating implant stability to bone structure. Clin Oral Implants Res.

[20] Turkyilmaz I, Aksoy U, McGlumphy EA (2008). Two alternative surgical techniques for enhancing primary implant stability in the posterior maxilla: a clinical study including bone density, insertion torque, and resonance frequency analysis data. Clin Implant Dent Relat Res.

[21] Alghamdi H, Anand PS, Anil S (2011). Undersized Implant Site Preparation to Enhance Primary Implant Stability in Poor Bone Density: A Prospective Clinical Study. J Oral Maxillofac Surg.

[22] Petrov SD, Xing Y, Khandelwal N, Drew HJ (2014). A novel technique for osteotome internal sinus lifts with simultaneous placement of tapered implants to improve primary stability. J Oral Implantol.

[23] Krafft T, Graef F, Winter W, Wichmann M, Karl M (2013). Use of osteotomes for implant bed preparation-effect on material properties of bone and primary implant stability. J Oral Implantol.

[24] Di Alberti L, Donnini F, Di Alberti C, Camerino M (2010). A comparative study of bone densitometry during osseointegration: piezoelectric surgery versus rotary protocols. Quintessence Int.

[25] Stacchi C, Vercellotti T, Torelli L, Furlan F, Di Lenarda R (2013). Preparation Techniques: Twist Drills versus Piezosurgery. A Single-Blinded, Randomized, Controlled Clinical Trial. Clin Implant Dent Relat Res.

[26] Preti G, Martinasso G, Peirone B, Navone R, Manzella C, Muzio G (2007). Cytokines and growth factors involved in the osseointegration of oral titanium implants positioned using piezoelectric bone surgery versus a drill technique: a pilot study in minipigs. J Periodontol.

[27] Cano J, Campo J (2012). Bone implant sockets made using three different procedures: a stability study in dogs. J Clin Exp Dent.

[28] Yoon HG, Heo SJ, Koak JY, Kim SK, Lee SY (2011). Effect of bone quality and implant surgical technique on implant stability quotient (ISQ) value. J Adv Prosthodont.

[29] Rodrigo D, Aracil L, Martin C, Sanz M (2010). Diagnosis of implant stability and its impact on implant survival: a prospective case series study. Clin Oral Implants Res.

